# Exchange Protein Directly Activated by cAMP 2 Enhances Respiratory Syncytial Virus-Induced Pulmonary Disease in Mice

**DOI:** 10.3389/fimmu.2021.757758

**Published:** 2021-10-18

**Authors:** Junping Ren, Wenzhe Wu, Ke Zhang, Eun-Jin Choi, Pingyuan Wang, Teodora Ivanciuc, Alex Peniche, Youwen Qian, Roberto P. Garofalo, Jia Zhou, Xiaoyong Bao

**Affiliations:** ^1^ Department of Pediatrics, University of Texas Medical Branch, Galveston, TX, United States; ^2^ Department of Chemistry, University of Houston Clear Lake, Clear Lake, TX, United States; ^3^ Department of Pharmacology and Toxicology, University of Texas Medical Branch, Galveston, TX, United States; ^4^ Department of Pathology, Roswell Park Cancer Institute, Buffalo, NY, United States; ^5^ Institute of Translational Sciences, University of Texas Medical Branch, Galveston, TX, United States; ^6^ Institute for Human Infections and Immunity, University of Texas Medical Branch, Galveston, TX, United States

**Keywords:** EPAC2, RSV, pulmonary disease, immune response, inflammation

## Abstract

Respiratory syncytial virus (RSV) is the most common cause of lower respiratory tract infection in young children. It is also a significant contributor to upper respiratory tract infections, therefore, a major cause for visits to the pediatrician. High morbidity and mortality are associated with high-risk populations including premature infants, the elderly, and the immunocompromised. However, no effective and specific treatment is available. Recently, we discovered that an exchange protein directly activated by cyclic AMP 2 (EPAC2) can serve as a potential therapeutic target for RSV. In both lower and upper epithelial cells, EPAC2 promotes RSV replication and pro-inflammatory cytokine/chemokine induction. However, the overall role of EPAC2 in the pulmonary responses to RSV has not been investigated. Herein, we found that EPAC2-deficient mice (KO) or mice treated with an EPAC2-specific inhibitor showed a significant decrease in body weight loss, airway hyperresponsiveness, and pulmonary inflammation, compared with wild-type (WT) or vehicle-treated mice. Overall, this study demonstrates the critical contribution of the EPAC2-mediated pathway to airway diseases in experimental RSV infection, suggesting the possibility to target EPAC2 as a promising treatment modality for RSV.

## Importance

Respiratory syncytial virus (RSV) is highly associated with bronchiolitis, pneumonia, and even death in infants younger than six months of age, the elderly, and the immunocompromised. Currently, no effective treatment or vaccine is available. In addition, many molecular mechanisms underlying RSV-induced lung diseases are not fully understood. Our study elucidated the roles of a protein called EPAC2, which is a target of a major second messenger cAMP, in RSV-induced pulmonary responses and associated disease. Therefore, our results are likely going to provide important insight into the development of new pharmacologic strategies against RSV infection.

## Introduction

Respiratory syncytial virus (RSV) is a significant respiratory pathogen, representing the most common cause to lower respiratory tract infection (LRTI) diseases in children, the elderly, and the immunocompromised. Together with other important viruses, such as influenza and human metapneumovirus, RSV exhibits strong seasonal activity and its medical burden usually exceeds that of influenza among young age groups (1, 2). It also contributes significantly to upper respiratory tract infection (URTI) resulting in a huge outpatient burden (3). Infection with RSV usually starts with the epithelium of the URT and then descends to the lower airways resulting in symptoms ranging from bronchiolitis to pneumonia, which always requires hospitalization. To date, there is no effective and specific treatment available against RSV infection, demonstrating the need to explore more disease mechanisms beyond current knowledge so that effective therapeutic approaches can be effectively integrated, designed, and developed.

Exchange proteins directly activated by cAMP (EPAC) are a fairly new receptor family of cyclic AMP (cAMP), compared with another major cAMP receptor family protein kinase A (PKA). Soon after the discovery of EPAC, they were found to be functionally important for various diseases including heart failure, cancer, neurological disorders, diabetes, and inflammation (4–8). They have two main isoforms: EPAC1 and EPAC2. The function of EPAC in viral infections is also emerging with associated regulatory mechanisms largely unknown (9–12). Unlike Ebola, Middle East respiratory syndrome coronavirus (MERS-CoV), and vesicular stomatitis virus (VSV), which activate the EPAC1-mediated pathway to favor virus invasion (9–11), we discovered that RSV uses EPAC2 to favor its survival in the airway epithelial cells (AECs) (12). EPAC2 deficient AECs, derived from both U/LRT, have a decrease in RSV replication and suppressed cellular inflammatory responses to RSV infection, compared with EPAC2 competent cells. By using an EPAC2-specific inhibitor, we also confirmed the role of EPAC2 in RSV infection in the AECs. However, the overall *in vivo* role of EPAC2 has not been investigated (12).

In this study, we demonstrated EPAC2 knockout (EPAC2-/-) mice had a significant decrease in body weight loss, basal airway hyperresponsiveness, pulmonary inflammation, and viral replication in response to RSV infection, compared with wild-type (WT) mice. Consistently, mice treated with EPAC2-specific inhibitor share similar disease symptoms, compared with the vehicle-treated mice. Other than its role in RSV-induced pulmonary inflammation, EPAC2 has been reported to be involved in inflammatory and remodeling processes induced by cigarette smoke (13), confirming its essential role in respiratory health. Taken together, our data indicate the role of EPAC2-mediated pathways in the pulmonary responses to RSV infection. Therefore, EPAC2 could serve as a novel potential therapeutic target to control both RSV replication and associated host inflammatory responses.

## Materials and Methods

### Cell Lines and RSV Preparation

RSV long strain was propagated in HEp-2 cells (ATCC, Manassas, VA) at 37°C and purified by sucrose gradient as described (14–16). Viral titer was determined by immunostaining in HEp-2 cells using biotin-conjugated goat anti-RSV primary antibody (Cat #: 7950-0104, Bio-rad, Hercules, CA), followed by the incubation with streptavidin peroxidase polymer (Cat#: S2438, Sigma-Aldrich, St Louis, MO), as previously described (14, 16).

### Mice

WT and EPAC2−/− mice (C57BL/6 background) are generous gifts from Ju Chen (University of California at San Diego) and grown under specific-pathogen-free conditions at the University of Texas Medical Branch (UTMB). Mice were anesthetized and infected intranasally (i.n.) with 107 plaque-forming units (p.f.u.) RSV diluted in Dulbecco’s PBS (D-PBS) (Invitrogen) as described (17–19). In the control group, mice were inoculated with an equivalent volume of sucrose diluted in D-PBS. All of the studies have been approved by the full board of the UTMB Institutional Animal Care and Use Committee, under protocol number 9001002J following the National Institutes of Health and University of Texas Medical Branch institutional guidelines for animal care.

We also studied the effect of EPAC2 inhibitor on the pulmonary responses to RSV. In brief, 10-week old BALB/c mice were infected with 3×10^6^ p.f.u. RSV and treated with EPAC2 inhibitor MAY0132 (N-(4-chloro-3-(trifluoromethyl) phenyl)-2,4,6-trimethylaniline, 20 mg/kg) or vehicle intranasally (i.n). We have carefully investigated the specificity of MAY0132 to inhibit EPAC2 (20, 21). MAY0132 was dissolved in ethanol to constitute 400 mg/mL stock and stored at -20°C. Before mice treatment, MAY0132 was freshly prepared in 10% PEG400-PBS and warmed to 37°C before treatment in mice. MAY0132 was given to mice five hours before RSV inoculation, then mice were treated with MAY0132 again at six hours postinfection (p.i) and days 1-3 p.i.

### Cytokine and Chemokine Quantification

To investigate the role of EPAC2 in regulating pulmonary innate responses, a multi-analytic profiling mouse (Cat#: M60-009RDPD) cytokine/chemokine kit from Bio-Rad (Hercules, CA) was used to measure RSV-induced cytokines/chemokines for bronchoalveolar lavage (BAL) samples from mice, uninfected or infected with RSV, according to the manufacturer’s instructions. Data were analyzed using the Multiplex Analyst Software from Bio-Rad.

### Quantitative RT-PCR

Total lung RNAs were extracted using TRIzol reagents (Thermo Fisher Scientific, Waltham, MA). qRT-PCR for viral replication or viral gene expression was performed using SYBR, as we previously described (12). The primers used to quantify the EPAC genes are available upon request.

### Bronchoalveolar Lavage Fluid Analysis

Total cells in the 50 μl of BALF were determined by trypan blue staining and the counting of viable cells was done by a hemocytometer. Differential cell counts were performed on cytocentrifuge preparations (Cytospin 3; Thermo Shandon, Pittsburgh, PA) stained with Wright-Giemsa stain (Thermo Fisher Scientific, Waltham, MA). A total of 200 cells per sample were counted by using light microscopy.

### Lung Pathology Analysis

The formalin-fixed lungs were embedded in paraffin, sectioned at a 4-μm thickness, and stained with hematoxylin and eosin or Masson’s trichrome. Microscopy was performed on a Nikon Eclipse Ti system, similar as described in (22).

### Airway Hyperresponsiveness

Basal AHR was assessed in unrestrained mice before or after infections at the days as indicated, using whole-body barometric plethysmography (Buxco, Troy, NY) to record enhanced pause (Penh), as previously described (17, 23). Penh value is to estimate airway resistance.

### Western Blot Analysis

Total lung lysates were prepared as previously described (24, 25). Proteins were then quantified with a protein quantification kit from Bio-Rad, followed by the fractionation using SDS-PAGE denaturing gels and protein transferring to polyvinylidene difluoride membranes. Membranes were blocked with 5% non-fat dried milk in TBS-Tween 20 and incubated with primary antibody, the biotin-conjugated goat anti-RSV, followed by the secondary anti-goat antibody (cat# sc-2354 from Santa Cruz, Santa Cruz, CA), according to the manufacturer’s instruction.

### FACS Analysis

Total lung cells were harvested at day 7 p.i. after mock or RSV infection as previously described (17, 19). Isolated cells were incubated with anti-FcγRII/FcγRIII mAb (24G2; BD Biosciences). For cell-surface marker staining, an aliquot of cells was stained with the following anti-mouse antibodies: anti-CD11c, anti-F4/80, anti-CD11b, and anti-Gr-1 (all from BD-Pharmingen). Samples were stained at 4°C in PBS with 1 % FBS and analyzed with a FACS Canto flow cytometer equipped with BD FACSDiva software (both from Becton Dickinson Immunocytometry Systems). Analysis was performed using WinMDI2.8 (Scripps).

### Statistical Analysis

One-way ANOVA analysis was performed, followed by Tukey’s *post hoc* test to determine significance. Mann-Whitney tests were used for nonparametric data. All data subjected to statistical analysis are means ± standard deviations (SD). A P value of <0.05 was considered significant.

## Results

### EPAC2 Promotes RSV-Induced Disease and Airway Obstruction *In Vivo*

**Figure 1:**
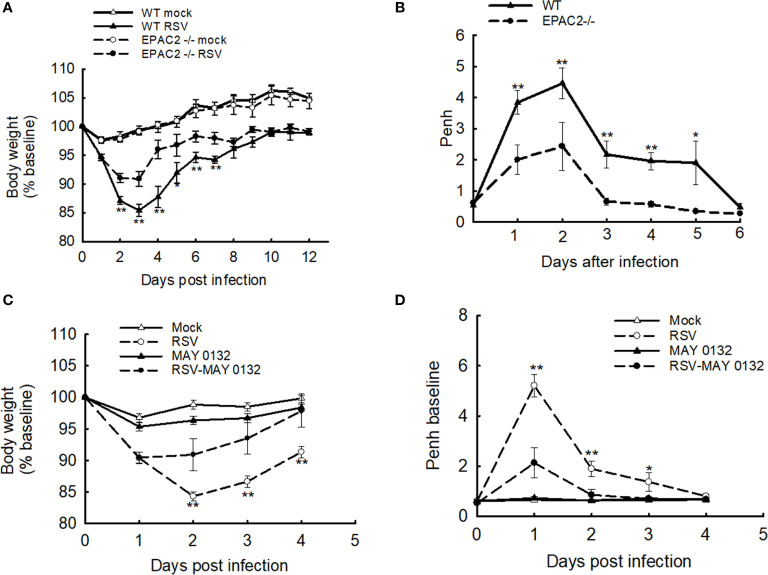
The effect of EPAC2 on disease severity in RSV-infected mice. **(A, B)** WT and EPAC2-/- mice (C57BL/6 background) were infected with 1×10^7^ pfu of RSV or appropriate volume of control vehicle intranasally (i.n.). **(A)** Bodyweight was monitored daily and expressed as a percentage of baseline weight. **(B)** Lung function in RSV-infected mice, either WT or EPAC2/-, at the days p.i. as indicated. Unrestrained, whole-body plethysmography (Buxco Electronicsm Inc. Sharon, CT) was used to measure the basal enhanced pause (Penh). The percentage of baseline weight and Penh values were presented as mean ± SEM (n = 4-5 mice/group, from four independent experiments). Asterisks indicate levels of significance, *P < 0.05 and **P < 0.01 for comparison to RSV-infected samples from EPAC2−/− mice. **(C, D)** 10-week old BALB/c mice were i.n. infected with 3×10^6^ p.f.u. RSV, followed by the treatment of EPAC2 inhibitor MAY0132 (20 mg/kg) or vehicle (PEG in PBS). MAY0132 was given to mice five hours before RSV inoculation, then mice were treated with MAY0132 again at six hours and days 1-4 p.i. Bodyweight **(C)** and basic Penh **(D)** were monitored daily. The percentage of baseline weight and Penh values were presented as mean ± SE (n = 4-5 mice/group, from three independent experiments). Asterisks indicate levels of significance, *P < 0.05 and **P < 0.01 for comparison to RSV-infected MAY0132-treated mice.

We have recently presented the idea that EPAC2 deficiency benefits the control of RSV replication and associated cellular inflammatory response in the AECs. To confirm its impact *in vivo*, we first used EPAC2 knockout (KO) mice to investigate whether any changes in disease presentation are associated with EPAC2 KO – following RSV infection. Consistent with the previous mouse model which showed a time-dependent body weight loss and airway obstruction of mice in response to RSV infection (23, 26), we also found that WT mice (C57BL/6 background) induced a significant body weight loss, which peaked at day three following the infection (**Figure 1A**) and experienced enhanced baseline and enhanced pause (Penh) with a peak at day two (**Figure 1B**). Compared with the infected WT mice, EPAC2 KO mice after the infection had lower body weight loss (**Figure 1A**) and attenuated enhancement in Penh (**Figure 1B**). Consistent with the impact of EPAC2 KO on RSV-induced disease and airway obstruction, we also found that the treatment of MAY0132, an EPAC2-specific inhibitor (27), attenuated RSV-induced body weight loss, starting at day two postinfection (p.i.), with a further clinical benefit at day three p.i. On day four p.i., the bodyweight loss showed no difference with that of mice without infection (**Figure 1C**). The treatment of MAY0132 also eased RSV-enhanced Penh, starting as early as day one p.i. and completely back to the normal basal level by day three p.i. (**Figure 1D**). The treatment of MAY0132 did not result in ruffled fur, suggesting minimal-to-no toxicity signs at the dose of 20 mg/kg. All these results support that EPAC2 deficiency ameliorates viral-induced disease and pulmonary function in response to RSV infection.

### EPAC2 Promotes Pulmonary Inflammation in RSV-Infected Mice

**Figure 2:**
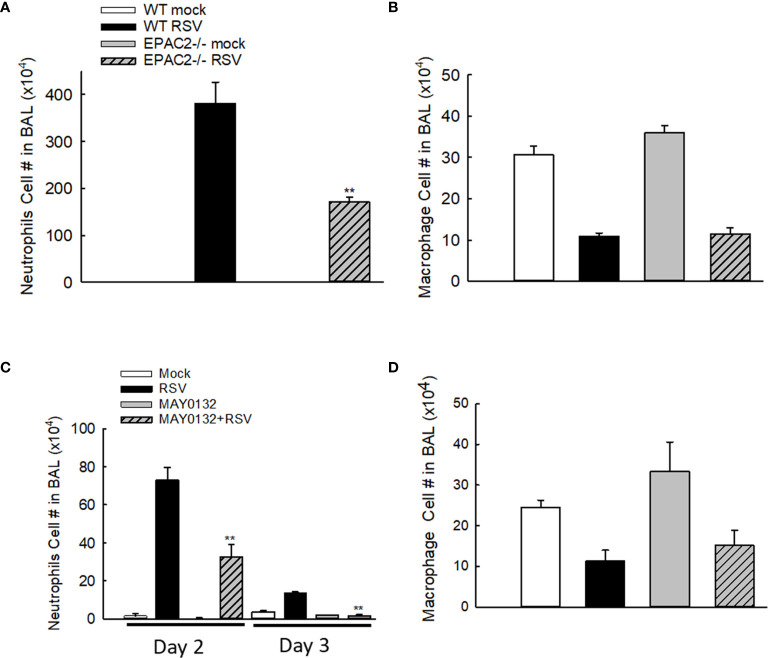
The impact of EPAC2 on the presence of immune cells in the BALF. **(A, B)** WT and EPAC2-/- mice were infected with RSV i.n., as described in **Figure 1**. Quantification of polymorphonuclear neutrophils **(A)** and macrophages **(B)** in BALF cytospins were carried out at day two p.i. **(C, D)** BALB/c mice, with/without MAY0132 treatment, were infected with RSV i.n., as described in **Figure 1**. Neutrophils **(C)** and macrophages **(D)** in BALF cytospins were quantified at day two or three p.i. n = 10-11 mice/group. The results are from three independent experiments. Asterisks indicate levels of significance, **P < 0.01 for comparison to RSV-infected samples from WT mice **(A)** or RSV-infected with MAY0132-treated mice **(C)**.

Neutrophils, whose recruitment to the airways usually peaks at 18-24 h p.i. (28), become the predominant inflammatory cells in BALF during the RSV infection (29). To investigate whether EPAC2 plays a role in modulating RSV-induced lung inflammation, we first compared the neutrophil infiltration in WT and EPAC2-/- mice. WT and EPAC2-/- mice were inoculated with RSV and the BALF samples were harvested at day two p.i. when a significant discrepancy in body weight loss and basal Penh started to show. The neutrophil recruitment into the airways was prevented in EPAC2-/- mice (**Figure 2A**). We found that at day three p.i., BAL neutrophil cells of RSV-infected mice, both WT and EPAC2-/-, went back to the basal level (data not shown). On day two p.i., RSV-infected mice had comparable BAL macrophages in WT and EPAC2-/- mice (**Figure 2B**). The impact of EPAC2 on pulmonary neutrophils and macrophages was also investigated by comparing their presence in BALF between RSV-infected mice treated with and without MAY0132. We found that MAY0132-treated mice had attenuated neutrophil infiltration than vehicle-treated mice at days two and three p.i. (**Figure 2C**). On day four p.i., the neutrophil cells in BAL, vehicle- or MAY0132-treated, went back to the basal level (data not shown). In conclusion, MAY0132 did not impact the BAL macrophage (**Figure 2D**).

### EPAC2 Contributes to the Production of Immune Mediators

**Figure 3:**
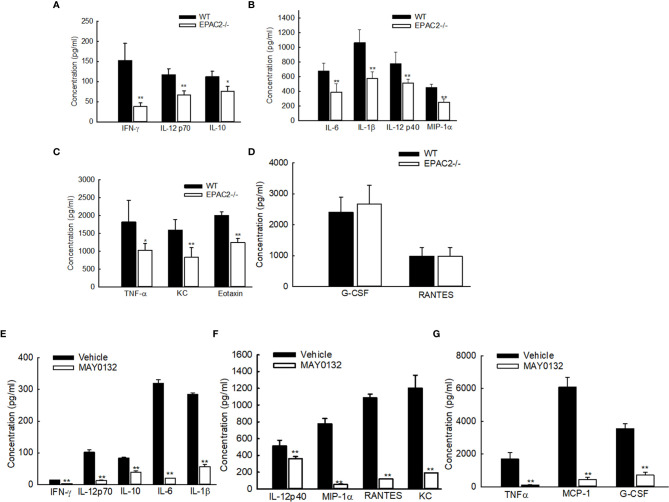
Impaired the cytokine/chemokine induction by EPAC2 deficiency. **(A–D)** WT or EPAC2-/- mice were sham infected or infected with RSV as described in **Figure 1**. The BAL fluid samples were collected at day two p.i., followed by cytokine/chemokines quantification using Bio-Plex Pro Mouse Cytokine 23-plex kit (Bio-rad, Cat #: M60009RDPD). The secretion is shown according to their absolute induction in the infected WT mice: 0-200 pg/ml **(A)**, 400-1400 pg/ml **(B)**, and more than 1500 pg/ml **(C)**. **(D)** unaffected immune mediators. n = 12 mice/group. The results, shown as mean ± SE, are from three independent experiments. Asterisks indicate levels of significance, *P < 0.05 and **P < 0.01 for comparison to RSV-infected samples from WT mice. **(E–G)** Mice were treated with MAY0132 or vehicle, RSV at the dose of 3×10^6^ pfu, or sham infected and harvested at day one postinfection to collect BALF samples to measure cytokines and chemokines by the multi-plex cytokine detection system. The mediators are shown in groups according to their absolute induction in the infected mice with vehicle treatment: 0-200 pg/ml **(E)**, 400-1400 pg/ml **(F)**, and more than 1500 pg/ml **(G)**. The results, shown as mean ± SE (*n* = 6 mice/group). Asterisks indicate levels of significance, *P < 0.05 and **P < 0.01 for comparison to RSV-infected vehicle-treated mice. G-CSF, granulocyte colony-stimulating factor; KC, neutrophil chemokine; MCP-1, monocyte chemoattractant protein-1; MIP-1, macrophage inflammatory protein-1; RANTES, regulated upon activation, normal T-cell expressed and secreted; TNF-α, tumor necrosis factor-α; IL, interleukin; IFN-γ, interferon-gamma.

To determine whether EPAC2 deficiency, either by knockout or the treatment of its inhibitor, affects RSV-induced immune mediator secretion, we also measured cytokine/chemokine levels in BAL samples collected at day two p.i., which is the induction peak of immune mediators following RSV infection (30). In RSV-infected mice, EPAC2 knockout significantly decreased the production of proinflammatory cytokines, such as IL-1α, IL-1β, IL-6, and TNF-α. Similar results were observed with the release of the chemokines regulated upon activation of MIP-1α, MIP-1β, MCP-1, and neutrophil chemokine KC (**Figures 3A–C**). The induction of RANTES and G-CSF was comparable in infected WT and EPAC2-/- mice (**Figure 3D**). We also found that UV-inactivated RSV significantly impaired cytokine/chemokine induction and the induction was comparable between infected WT- and EPAC2-/- mice (**Figure S1**), suggesting the EPAC2-mediated innate response is replication-dependent.

Consistent with the attenuated secretion of cytokines/chemokines in RSV-infected EPAC2-/- mice, the treatment of MAY0132 resulted in a suppressed inflammatory mediator induction, as well (**Figures 3E–G**).

### EPAC2 Deficiency Reduces RSV Replication

**Figure 4:**
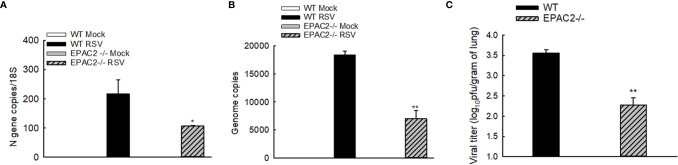
Inhibited RSV replication and pulmonary inflammatory responses by EPAC2 knockout. WT or EPAC2-/- mice were sham infected or infected with RSV as described in **Figure 1**. **(A, B)**. On day four p.i., the total RNAs of lungs were prepared, followed by real-time PCR to quantify RSV genome copies **(A)** and RSV N gene copies **(B)**. Lungs were also harvested on day five p.i. The infectious particles were quantified in Hep2 cells by immune staining **(C)**. The results, shown as mean ± SE (*n* = 6-12 mice/group). Asterisks indicate levels of significance, *P < 0.05 and **P < 0.01 for comparison to RSV-infected WT mice.

We have recently shown that EPAC2 deficiency leads to RSV replication suppression in AECs (12). To determine whether EPAC2 knockout alters RSV replication in the lung, we used qRT-PCR and plaque assay to determine the changes in viral genome/gene copies and infectious viral particles, respectively, by EPAC2 knockout. Compared with WT mice, EPAC2-/- mice exhibited significantly less RSV N gene and genome copies number at day four p.i. (**Figures 4A, B**). In addition, the lung viral titer of EPAC2-/- at day five p.i was also significantly less than that of WT mice (**Figure 4C** **)**. Days four and five are usually the peak of the replication in the experimental models of RSV (26, 31).

**Figure 5:**
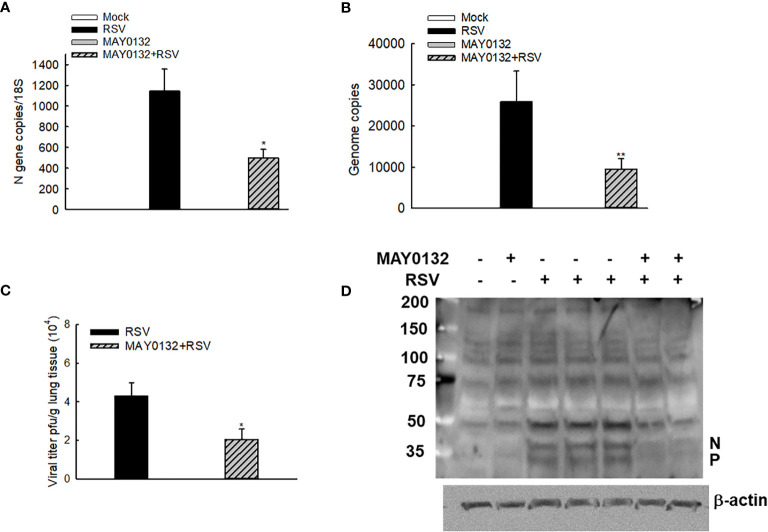
MAY0132-suppressed RSV replication. Mice, treated with MAY0132 or vehicle, were infected RSV at the dose of 3×10^6^ pfu, or sham infected, and the lungs were harvested at day four p.i. **(A)** The total lung RNAs were prepared, followed by real-time PCR to quantify the viral genome. **(B)** Viral N gene copies were also determined by real-time PCR. **(C)** Lung infectious viral particles were titrated on Hep2 cell monolayers by immune staining assays. **(D)** The total lungs were also harvested to determine the viral proteins by Western blot. n = 5-6 mice in each group. The results are from two independent experiments. Asterisks indicate levels of significance, *P < 0.05 and **P < 0.01 for comparison to RSV-infected compound untreated mice.

The mice were also treated with 20mg/kg of MAY0132 or control vehicle as described in “*Methods*”, and lungs were harvested at day four p.i. We found that a decrease of N and virus genome copies in the lungs were observed in MAY0132-treated mice, compared to the infected vehicle-treated mice (**Figures 5A, B**). A significant reduction of RSV peak titer was observed in MAY0132-treated animals (**Figure 5C**). The reduction of viral particles in the lungs by MAY0132 was also confirmed by a Western blot assay using an anti-RSV antibody. Significantly fewer viral proteins in the lungs were observed in MAY0132-treated animals than vehicle animals (**Figure 5D**). The results of these experiments showed that the administration of MAY0132 was effective in reducing RSV viral replication in the lung.

### The Treatment of EPAC2 Inhibitor Lessens RSV-Induced Pathogenesis

**Figure 6:**
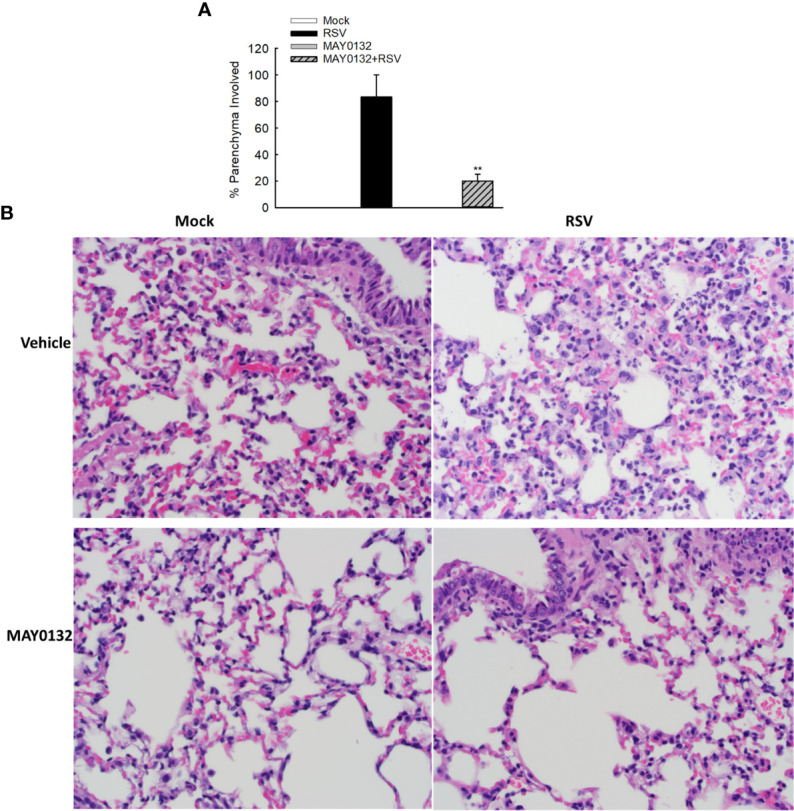
The contribution of EPAC2 to the initial lung inflammation after infection. Mice, treated with or without MAY0132, was sham infected or infected with RSV. Lungs were collected on day four after infection, followed by formalin fixation for slide preparation. Representative hematoxylin and eosin-stained lung tissue sections from the indicated treatment. **(A)** The bar graph represents mean ± SE (*n* = 3–4 mice/group). **P <  0.001 compared with vehicle/RSV mice. **(B)** The representative pathology slides are shown as described.

To further confirm the efficacy of MAY0132 in suppressing RSV-induced innate inflammation *in vivo*, we used histological assessment to exam the effect of EPAC2 inhibitor on lung pathology. Alveolar neutrophils and interstitial lymphocytes infiltrate at day four p.i. was less in MAY0132-treated mice than in control mice. Compared to control vehicle treatment, less inflammatory cells and structural damages in lungs were impacted by RSV with MAY0132 treatment (**Figure 6**), supporting the protective feature of MAY0132 on RSV-induced lung pathogenesis and highlighting a potential pharmaceutical intervention to treat RSV.

## Discussion

RSV is the leading cause of severe LRTI in children. It is currently estimated that 33.1 million episodes of RSV-associated LRTI lead to about 3.2 million hospital admissions and 59,600 in-hospital deaths of children younger than five years old globally. In addition, in-hospital deaths, due to RSV-caused LRTI, contribute to about 45% of hospital admitted patients younger than six months old, demonstrating a considerable burden of RSV infection on health-care services (32, 33). Palivizumab, although available for preventing RSV-associated hospitalizations, is not very cost-effective and is mainly limited to selected high-risk infants for the first RSV season (34). Other than palivizumab, there is no other prophylactic method. In addition, no effective treatment besides supportive measures is available. Herein, we demonstrated that EPAC2 controls pulmonary responses, viral replication, and the overall diseases in animal models of RSV infection, supporting EPAC2 to be a new potential target in the development of new treatment modalities for RSV.

As a recently discovered family of cAMP sensors, EPAC has been found to have two major isoforms: EPAC1 and EPAC2. In humans, both EPAC1 and EPAC2 have ubiquitous tissue distribution, with no expression of EPAC1 and EPAC2 in ovarian tissue and oral mucosa, respectively. Compared with EPAC1, whose signalosomes have been extensively studied, current knowledge on the significance/function mechanisms of EPAC2 is very limited, but discovered cellular interacting partners of EPAC2 are very distinct from those of EPAC1 (35). EPAC2 has been demonstrated to associate with Rab3-interacting molecule 1 (Rim1), Rim2, Piccolo, and synaptosome-associated protein of 25 kDa (SNAP-25) to mediate cAMP-induced PKA-independent exocytosis, which is important for regulating neurotransmission and insulin secretion (36, 37). EPAC2 also interacts with the cytoskeleton, controlling vesicle trafficking (38). Currently, we do not know what EPAC2’s partners are in mice to regulate RSV replication and innate pulmonary responses to RSV infection.

In this study, we also found that EPAC2 deficiency led to less hyperresponsiveness of infected mice (**Figure 1**). Penh is highly related to airway smooth muscle relaxation, therefore, it is possible that EPAC2 deficiency, by either KO or inhibitor treatment, helps smooth muscle relaxation during the infection. However, it has been previously shown that the reduction of EPAC by siRNA was unable to prevent cell stiffness (39). In addition, EPAC activation is not involved in smooth muscle relaxation mediated by dopamine (40). Therefore, the EPAC2-regulated airway hyperresponsiveness in the context of RSV may not result from the changes in muscle relaxation. Airway hyperresponsiveness is also highly related to pulmonary inflammation. EPAC2 has been previously reported to regulate cigarette smoke (CS)-induced inflammatory responses, characterized by the change of immune cells and cytokine release. Compared to wild-type mice exposed to CS, the number of total inflammatory cells, macrophages, and neutrophils and total IL-6 release is lower in EPAC2 KO mice (13). The phospholipase-Cϵ (PLCϵ) is the effector of EPAC. Suppressed neutrophils and IL-6 are also observed in PLCϵ-/- mice, supporting the importance of EPAC2/PLCϵ axis in mediating airway inflammation. Whether alleviated RSV-induced hyperresponsiveness by EPAC2 KO or MAY0132 treatment is through EPAC2/PLCϵ/IL-6/neurophils signaling axis is currently unknown, but we did observe EPAC2 deficiency-caused suppression of IL-6 induction and pulmonary neutrophil. Decreased neutrophil in the infected lung is indeed still detectable at day seven p.i. when most cytokines/chemokines go back to the basal level and infectious particles are not detectable anymore (**Figure S2**), highlighting the role of EPAC2 in controlling RSV-induced inflammation.

We recently discovered that EPAC2 deficiency leads to the suppression of RSV fusion protein translation and viral gene transcription and genome replication in the airway epithelial cells. In the meanwhile, the deficiency also inhibits cellular innate responses to RSV infection in both human lung epithelial cells and mouse embryonic fibroblasts (MEFs), likely through attenuating RSV-activated p50 activation (12). Some viral-induced immune mediators are inflammatory, therefore, detrimental to airway structure. Some are beneficial as they serve as antiviral molecules. In the case of EPAC2 deficiency, although suppressed cytokine/chemokine induction may favor RSV replication, direct inhibition of viral replication by EPAC2 inhibitor or KO could make the impact of cytokine/chemokine induction on viral replication relatively unimportant in this case. In contrast, decreased cytokine/chemokine induction, either directly by EPAC2 deficiency or indirectly resulting from suppressed viral replication, likely contributed to the prevention of inflammatory immune cell infiltration, hyperresponsiveness, and bodyweight loss, making the EPAC2 inhibitors therapeutically promising.

Overall, the development of inhibitors specific to cAMP and its effector molecules has greatly helped researchers to identify essential cAMP-related signaling pathways under various conditions, including viral infection. For example, the inhibitors facilitated the discovery showing that macrophage and T cells respectively use cAMP/PKA and cAMP/EPAC/Rap1 signaling to control human immunodeficiency virus type 1 replication (41, 42). In combination with the gene knockdown and knockout, we also used an EPAC2-specific inhibitor to define EPAC2 as an essential and novel determinant of AECs to control RSV replication and associated inflammatory responses (12). The EPAC isoform in RSV infection is distinct from the isoform reported to be involved in other viral infections. While EPAC1 is essential for promoting Ebola, MERS-CoV, and VSV entry or replication (9–11), herein, we confirmed the role of EPAC2 in RSV infection *in vivo*. EPAC2 is not only critical in RSV replication, but also pulmonary responses and disease pathogenesis. Compound modification is currently carried out to make the inhibitor more water-soluble and potent, similar as described (43). Developing compounds that can control both replication and inflammation is ideal as accumulating data support that both direct damages from viral replication and the host immune-inflammatory response contribute to RSV-induced respiratory disease, although their relative weight remains controversial (44–46).

In conclusion, we have shown that pulmonary EPAC2 significantly impacts host responses and viral replication in two mice models of RSV infection, supporting EPAC2 could be a promising therapeutic target for RSV infection. In the future, we will focus on developing potent EPAC inhibitors against viral infections, studying the antiviral spectrum of EPAC inhibitors, and the role of EPAC2 in RSV-caused immune cell responses.

## Data Availability Statement

The original contributions presented in the study are included in the article/**Supplementary Material**. Further inquiries can be directed to the corresponding author.

## Ethics Statement

The animal study was reviewed and approved by IACUC Committee, University of Texas Medical Branch, Galveston, Texas, 77555.

## Author Contributions

XB wrote the manuscript. JR, E-JC, WW, KZ, PW, TI, AP, RG, and XB contributed to experimental design/performance and data analysis. YQ, pathological slide reading. PW and JZ synthesized EPAC2-specific inhibitors. XB is responsible for project oversight. All authors contributed to the article and approved the submitted version.

## Funding

This work was supported by grants from the NIH R01 AI116812 and R21 AG069226, and FAMRI Clinical Innovator Award 160020 to XB and NIH AI062885 to RG. JZ is partly supported by John D. Stobo, M. D. Distinguished Chair Endowment Fund at UTMB.

## Conflict of Interest

The authors declare that the research was conducted in the absence of any commercial or financial relationships that could be construed as a potential conflict of interest.

## Publisher’s Note

All claims expressed in this article are solely those of the authors and do not necessarily represent those of their affiliated organizations, or those of the publisher, the editors and the reviewers. Any product that may be evaluated in this article, or claim that may be made by its manufacturer, is not guaranteed or endorsed by the publisher.

## Supplementary Material

Supplementary Figure 1 The EPAC2-mediated pulmonary innate response is replication-dependent. Mice, WT or EPAC2-/-, were infected with UV-inactivated RSV at the dose of 10^7^ pfu, or sham infected. On day 2 p.i., The BAL fluid samples were collected at day two p.i., followed by cytokine/chemokines quantification using Bio-Plex Pro Mouse Cytokine 23-plex kit. Immune mediators, which are not displayed, were not detectable by kit. n = 10 mice/group. The results, shown as mean ± SE, are from three independent experiments.

Supplementary Figure 2 Effect of EPAC2 on lung neutrophiles and macrophages at day seven p.i. Mice, WT or EPAC2-/-, were infected with RSV at the dose of 10^7^ pfu, or sham infected. At day seven p.i., pulmonary neutrophils **(A, B)** and macrophages **(C, D)** were quantified by FACS. The cell percentage **(A, C)** and total cell numbers **(B, D)** are presented as mean ± SEM, n = 6 in each group from two independent experiments. Asterisks indicate levels of significance, **P* < 0.05 for comparison to RSV-infected WT mice.
