# Supplementary figures and images for: Exchange Protein Directly Activated by cAMP 2 Enhances Respiratory Syncytial Virus-Induced Pulmonary Disease in Mice
Source: Front Immunol. 2021 Oct 18;12:757758. doi: 10.3389/fimmu.2021.757758 (PMC8558466; doi:10.3389/fimmu.2021.757758)

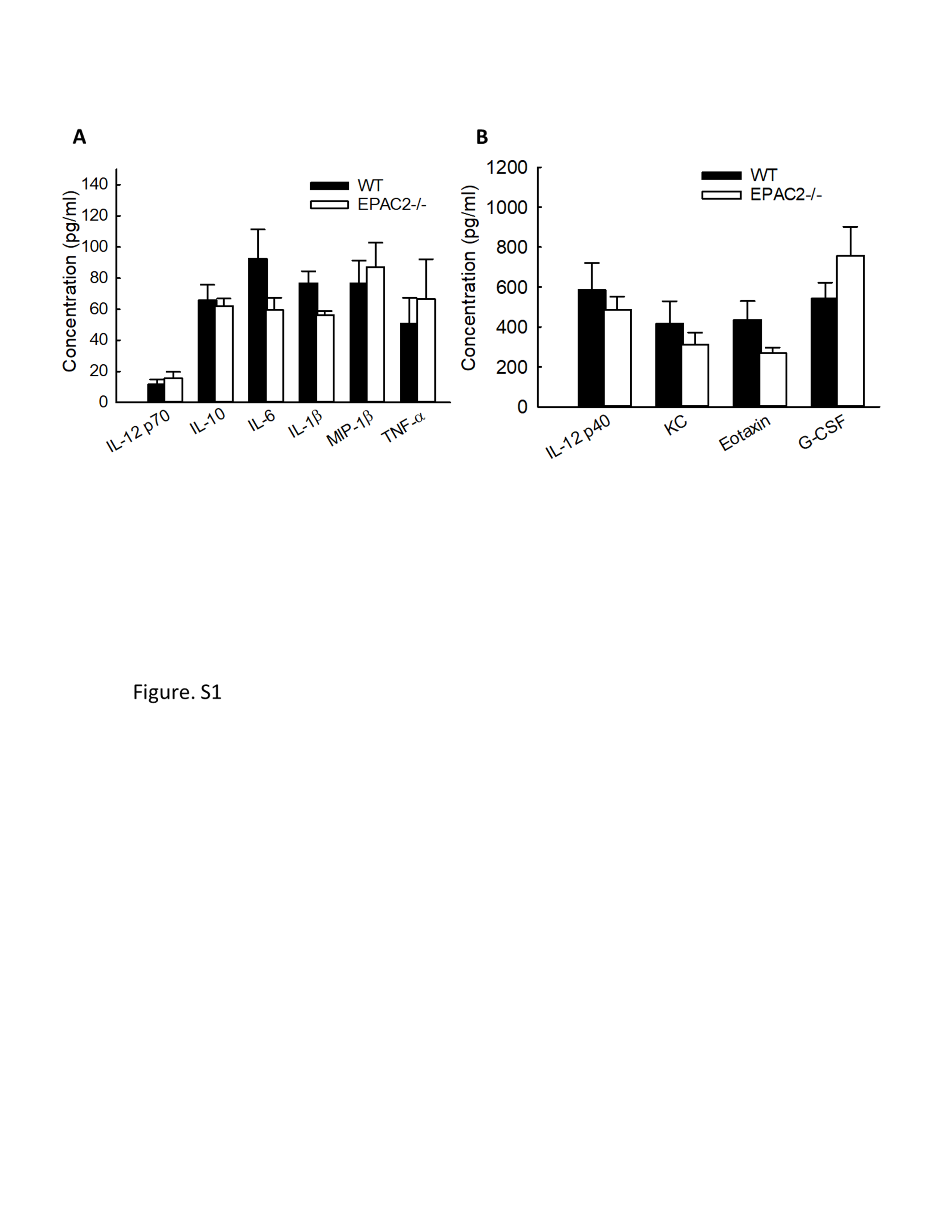

Supplement: Supplementary Figure 1 — The EPAC2-mediated pulmonary innate response is replication-dependent. Mice, WT or EPAC2-/-, were infected with UV-inactivated RSV at the dose of 107 pfu, or sham infected. On day 2 p.i., The BAL fluid samples were collected at day two p.i., followed by cytokine/chemokines quantification using Bio-Plex Pro Mouse Cytokine 23-plex kit. Immune mediators, which are not displayed, were not detectable by kit. n = 10 mice/group. The results, shown as mean ± SE, are from three independent experiments. [file Image_1.tif]

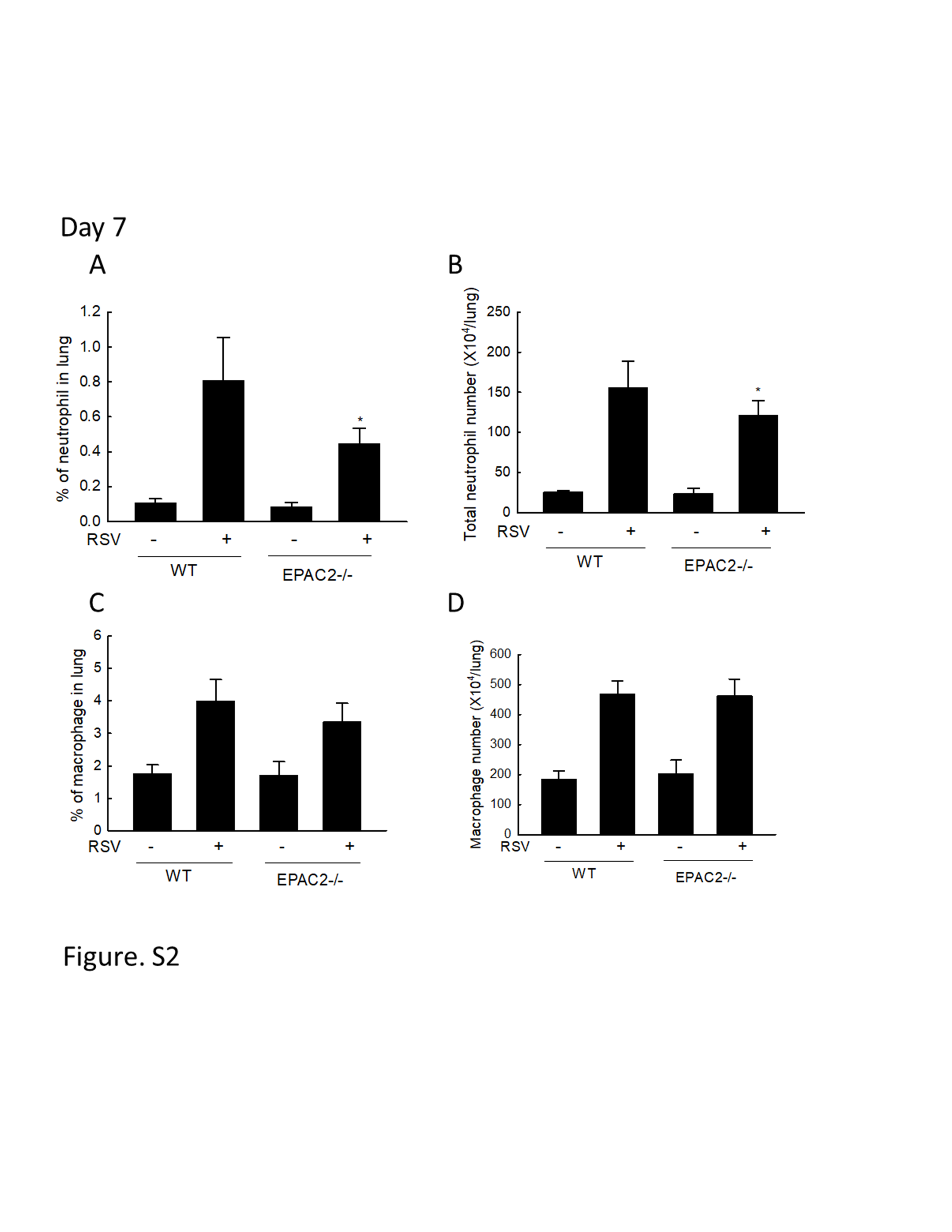

Supplement: Supplementary Figure 2 — Effect of EPAC2 on lung neutrophiles and macrophages at day seven p.i. Mice, WT or EPAC2-/-, were infected with RSV at the dose of 107 pfu, or sham infected. At day seven p.i., pulmonary neutrophils (A, B) and macrophages (C, D) were quantified by FACS. The cell percentage (A, C) and total cell numbers (B, D) are presented as mean ± SEM, n = 6 in each group from two independent experiments. Asterisks indicate levels of significance, *P < 0.05 for comparison to RSV-infected WT mice. [file Image_2.tif]
